# 179. INSPIRE Trial: A 92-Hospital Cluster Randomized Trial of INtelligent Stewardship Prompts to Improve Real-time Empiric Antibiotic Selection versus Routine Antibiotic Selection Practices for Patients with Skin and Soft Tissue Infections

**DOI:** 10.1093/ofid/ofae631.059

**Published:** 2025-01-29

**Authors:** Shruti K Gohil, Edward J Septimus, Ken Kleinman, Neha Varma, Kenneth E Sands, Amarah Mauricio, Taliser R Avery, Selsebil Sljivo, Risa Rahm, Kaleb Roemer, William S Cooper, Laura E McLean, Naoise G Nickolay, Russell Poland, Robert A Weinstein, Samir M Fakhry, Jeffrey Guy, Julia Moody, Micaela H Coady, Kimberly Smith, Brittany Meador, Allison Froman, Katyuska Eibensteiner, Mary K Hayden, David W Kubiak, Chenette Burks, L Hayley Burgess, Karla Miller, Michael S Calderwood, Jonathan B Perlin, Michael Cuffe, Richard Platt, Susan Huang

**Affiliations:** University of California, Irvine, Irvine, CA; Texas A&M University System Health Science Center, Houston, Texas; University of Massachusetts Amherst, Amherst, Massachusetts; Department of Population Medicine, Harvard Pilgrim Health Care Institute, Boston, Massachusetts; HCA Healthcare, Nashville, Tennessee; Division of Infectious Diseases, University of California, Irvine School of Medicine, Irvine, California; Department of Population Medicine, Harvard Pilgrim Healthcare Institute, Harvard Medical School, Boston, Massachusetts; Department of Population Medicine, Harvard Pilgrim Healthcare Institute, Boston, Massachusetts; HCA Healthcare, Nashville, Tennessee; HCA Healthcare, Nashville, Tennessee; HCA Healthcare, Nashville, Tennessee; HCA Healthcare, Nashville, Tennessee; HCA Healthcare, Nashville, Tennessee; HCA Healthcare, Nashville, Tennessee; Rush Medical College, Chicago, Illinois; HCA Healthcare, Nashville, Tennessee; HCA Healthcare, Nashville, Tennessee; HCA Healthcare, Nashville, Tennessee; Department of Population Medicine, Harvard Pilgrim Healthcare Institute, Boston, Massachusetts; HCA Healthcare, Nashville, Tennessee; HCA Healthcare, Nashville, Tennessee; Department of Population Medicine, Harvard Pilgrim Healthcare Institute, Boston, Massachusetts; Department of Population Medicine, Harvard Pilgrim Healthcare Institute, Boston, Massachusetts; Rush University Medical Center, Chicago, IL; Brigham & Women's Hospital, BOSTON, Massachusetts; HCA Healthcare, Nashville, Tennessee; HCA Healthcare, Nashville, Tennessee; HCA Healthcare, Nashville, Tennessee; Dartmouth Hitchcock Medical Center, Hanover, NH; The Joint Commission, Washington, District of Columbia; HCA Healthcare, Nashville, Tennessee; Department of Population Medicine, Harvard Pilgrim Health Care Institute and Harvard Medical School, Boston, MA, USA, Boston, Massachusetts; University of California, Irvine School of Medicine, Irvine, California

## Abstract

**Background:**

Up to 40% of hospitalized patients receive extended-spectrum (ES) antibiotics despite low risk of multidrug-resistant organism (MDRO) infection, increasing the risk for adverse effects and future resistance. We evaluated whether computerized physician order entry (CPOE) prompts providing patient-specific MDRO risk estimates could reduce ES antibiotic use compared to routine stewardship practices in patients hospitalized with skin and soft tissue (SST) infections.

**Figure:**
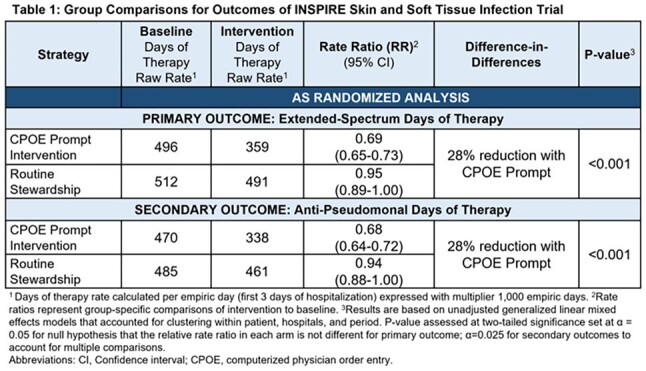

**Methods:**

This 92-hospital cluster-randomized trial compared CPOE prompts providing patient-specific absolute risk estimates for MDRO SST infection and recommending standard-spectrum antibiotics for risk < 10% vs. routine stewardship. Trial population: adults treated with antibiotics for SST infection in non-ICUs in the first 3 days of admission (empiric period). Prompts were triggered if ES antibiotics were ordered. Trial periods: 12-month Baseline (Jan 2019-Dec 2019); 5-month Phase-in (Aug 2022–Dec 2022); 12-month Intervention (Jan 2023-Dec 2023). Primary outcome: ES antibiotic days of therapy (ES-DOT) per patient per empiric day; secondary outcome was anti-pseudomonal DOT per empiric day. Unadjusted, as-randomized analyses used (1) generalized linear mixed effects models to assess differences in ES-DOT rates across intervention and baseline periods between groups, clustering by patient, hospital, and period and (2) proportional hazards models to assess safety outcomes: days to ICU transfer and hospital LOS.

**Figure:**
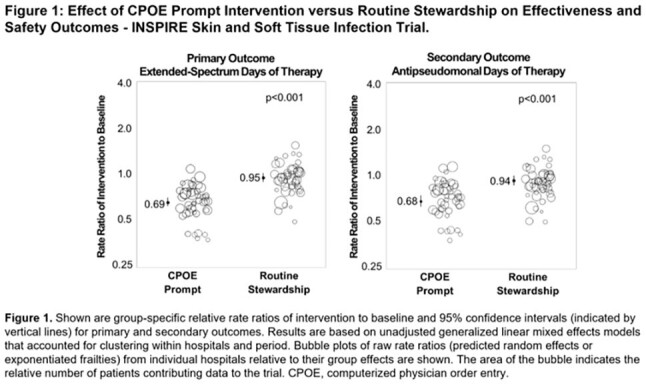

**Results:**

We randomized 92 hospitals in 15 states. Across the baseline and intervention periods there were 60,654 and 57,655 non-ICU patients with skin and soft tissue infection in the routine and CPOE prompt groups, respectively. The CPOE prompt group had a 28% reduction in ES-DOT compared to routine care (rate ratio 0.72 [95% CI 0.67-0.79], p< 0.001). Anti-pseudomonal DOT was reduced by 28% (Table, Figure 1) without significant differences in LOS or ICU transfers.

**Conclusion:**

INSPIRE CPOE prompts providing patient-specific MDRO risk estimates recommending standard spectrum antibiotics in low risk patients significantly reduced empiric ES prescribing in adults admitted with skin and soft tissue infection.

**Disclosures:**

**Ken Kleinman, ScD**, Xttrium Laboratories: Conducting studies in which participating hospital patients received contributed antiseptic products outside the submitted work **Richard Platt, MD, MSc**, GlaxoSmithKline: Contract to academic department|Janssen: Contract to academic department|Pfizer: Contract to academic department **Susan Huang, MD, MPH**, Xttrium Laboratories: Conducting studies in which participating hospital patients received contributed antiseptic products outside the submitted work

